# Determination of patient prognosis based on unruptured basilar artery aneurysm morphology using a novel dynamic prediction model

**DOI:** 10.3389/fneur.2026.1695091

**Published:** 2026-05-14

**Authors:** Wei Wang, Shilin Liu, Yifan Fu, Xiaoxuan Li, Minghao Xu, Xiaochan Xu, Tao Jiang

**Affiliations:** 1 Department of Emergency, The Second Affiliated Hospital of Anhui University of Traditional Chinese Medicine, Hefei, Anhui, China; 2 Department of Neurosurgery, The First Affiliated Hospital of Anhui Medical University, Hefei, China; 3 Anhui Public Health Clinical Center, Hefei, China

**Keywords:** basilar artery aneurysm, endovascular embolization, nomogram, survival prediction, vascular morphology

## Abstract

**Introduction:**

The study aimed to develop a novel predictive system to assess the clinical survival of patients with basilar artery aneurysms (BAAs) following endovascular embolization by analyzing aneurysm and peripheral vessel morphologies.

**Methods:**

Fifty-eight BAA patients who underwent endovascular embolization were retrospectively studied, with a median follow-up of 17.5 months. A total of nine deaths (15.5%) occurred during the follow-up period. Due to the limited number of outcome events, we adopted a conservative modeling strategy that involved using the least absolute shrinkage and selection operator (LASSO)-penalized Cox regression for variable selection, followed by multivariable Cox regression to construct the prognostic model. An online dynamic nomogram was developed to predict survival. Model discrimination was evaluated using the concordance index (C-index) and time-dependent receiver operating characteristic (ROC) curves. Internal validation was performed using bootstrap resampling with calibration curves, and clinical utility was assessed using decision curve analysis (DCA).

**Results:**

LASSO-penalized Cox regression identified two independent prognostic factors—basilar artery (BA) diameter and aneurysm neck—which were subsequently confirmed by multivariable Cox regression (BA: HR = 2.12, 95% CI: 1.01–4.45, *p* = 0.047; aneurysm neck: HR = 2.29, 95% CI: 1.04–6.23, *p* = 0.003). The model demonstrated acceptable discrimination, with a C-index of 0.805. Calibration curves showed good agreement between predicted and observed survival rates. The DCA demonstrated superior net benefit across a broad range of threshold probabilities (0–100%), with a net reduction of more than 80 unsuccessful procedures per 100 patients at a threshold of 85%.

**Conclusion:**

This morphology-based, online, dynamic nomogram serves as a practical tool for predicting survival in BAA patients after endovascular embolization. However, given the limited sample size and the number of events, the findings should be considered exploratory, and external validation in larger cohorts is warranted.

## Introduction

Ruptured intracranial aneurysms are an important cause of stroke, and basilar artery (BA) aneurysms account for approximately 5% of intracranial aneurysms (1). Patients with ruptured BA aneurysms have a higher incidence of disability and mortality due to their deep location and proximity to critical structures such as the brainstem (2, 3). Accordingly, managing basilar artery aneurysms (BAAs) holds critical significance. In cases of basilar artery aneurysms, the dominance of the vertebral artery can affect the hemodynamics of the vertebrobasilar arteries. At the same time, with aging, the basilar artery tends to meander to one side, which may have an effect on the occurrence and distribution of aneurysms. This suggests that the occurrence and development of aneurysms are closely related to local hemodynamic factors (2). In addition, previous studies have shown that the width of the apical angle at the bifurcation of the anterior cerebral artery (ACA), middle cerebral artery (MCA), and basilar artery (BA) is significantly greater compared to aneurysm-free bifurcations (3–5). In the treatment of patients with unruptured basilar artery aneurysms, the risk of aneurysm rupture over time is often clinically compared with the risk of surgery. This assessment, which incorporates patients’ clinical and imaging data, helps determine the most appropriate strategy—whether surgical treatment, interventional treatment, or watchful waiting. The objective is to guide patients toward the treatment pathway that presents the lowest overall risk. Endovascular embolization, known for its minimally invasive and precise characteristics, has gradually become the treatment of choice for BA aneurysms in recent years (6–8). However, the majority of studies conducted in this period have mainly analyzed risks by correlating unifactorial or multifactorial variables based on the morphology and hemodynamics of the aneurysm, and there is no recognized predictive model for assessing survival after endovascular embolization of basilar artery aneurysms (9). In recent years, advanced imaging and machine learning techniques have been increasingly used to predict intracranial aneurysm rupture risk and treatment outcomes, including radiomics-based models and deep learning approaches (10–12). However, the majority of these models focus on rupture risk rather than post-interventional survival, and few have been translated into user-friendly, web-based tools for real-time clinical use.

In addition, the majority of previous studies have only developed static prognostic prediction models. This limitation prevents timely clinical access to the predictive results of relevant models based on different patient-specific indicators (13, 14). Previous studies have shown that reliable and accurate predictive models can be constructed based on preoperative clinical and imaging data (15). To verify this hypothesis, we constructed a morphological model for basilar artery aneurysms by analyzing preoperative clinical and imaging data from patients. We used Cox proportional hazards regression analysis to develop a dynamic nomogram prediction model, which we expect will serve as a useful reference for clinicians in selecting the optimal treatment plan.

## Materials and methods

### Demographics

We retrospectively analyzed imaging data obtained from 58 consecutive patients who underwent brain computed tomographic angiography (CTA) at the North District of the First Affiliated Hospital of Anhui Medical University between 2019 and 2022. The inclusion criteria were as follows: (1) diagnosis of basilar artery aneurysm based on preoperative CTA imaging, (2) age ≥ 18 years, (3) endovascular embolization as the primary treatment, and (4) availability of complete preoperative imaging data for morphological analysis. The exclusion criteria were as follows: (1) prior surgical or endovascular intervention for intracranial aneurysms, (2) concomitant intracranial tumors or other malignancies, (3) incomplete clinical or imaging data, and (4) loss to follow-up during the study period. None of the patients received any invasive interventional treatments, such as open aneurysm clamping or vascular bypass with aneurysm isolation, before surgery. Postoperative survival was followed up immediately after surgery, prior to discharge, and at regular intervals thereafter. Information was collected via telephone follow-up, with a median follow-up duration of 17.5 months. The demographic characteristics of the study population are shown in Table 1. The study was approved by the Ethics Committee of the First Affiliated Hospital of Anhui Medical University, North District, Anhui, China (Approval no. PJ-YX2023-013). Informed consent was waived. In addition, as this was a retrospective analysis, there was no interference with patients’ diagnostic or therapeutic processes.

**Table 1:** Demographic characteristics of patients undergoing interventional embolization of basilar aneurysms.

Characteristic	Value
Sex	
Female	38
Male	28
Age	65 (55.2–72)
Hypertension	14
Diabetes	3
Smoking	15
Alcohol abuse	5
Angle of arterial bifurcation	
a1	135.05 (110.53–181.05)
a2	94.42 (65.81–109.06)
a3	126.66 (103.61–136.78)
LSR	1.29 (1.09–1.46)
Vessel diameter	
D1	1.75 (1.27–2.33)
D2	2.45 (1.89–2.96)
BA	3.37 (3.05–3.79)
DR	0.78 (0.7–0.91)
Arterial aneurysm parameters	
Aneurysm neck	5.09 (3.94–6.23)
Maximum diameter	4.27 (3.04–6.14)
Width	4.02 (2.96–5.27)
Height	4.03 (2.82–5.62)
Incidence angle	54.61 (24.77–92.69)
Volume	17.77 (9.64–26.83)

### Construction of three-dimensional models

We referred to the method proposed by Gao et al. (16) and constructed a 3D model based on patients’ CTA data using Materialise’s Interactive Medical Image Control System (Mimics) software. All 3D reconstructions were constructed using Materialise’s Interactive Medical Image Control System (Mimics, version 21.0; Materialise NV, Leuven, Belgium), based on preoperative CTA DICOM data. The reconstruction process followed these steps:

(1) Image import and thresholding: DICOM images were imported into Mimics. A bone tissue threshold (226–3,071 Hounsfield units) was applied to isolate vascular structures. The region of interest (the vertebrobasilar system) was manually selected.
(2) 3D region growing: The “Region Growing” tool was used to extract the continuous vascular tree from the thresholded mask, ensuring the separation of the basilar artery and its branches from the adjacent bone.
(3) 3D model generation: The extracted mask was converted into a 3D object using the “Calculate Part” function with standard smoothing parameters (smoothing factor = 0.5, iterations = 5) to reduce surface irregularities while preserving anatomical detail.
(4) Morphological measurements: All measurements were performed on the 3D models using Mimics measurement tools. (1) Angles (bifurcation apex angle a1, small lateral angle (LA) a2, large lateral angle a3, and incidence angle) were measured using the “Angle” tool, which calculates the angle between three points placed along vessel centerlines or vessel walls. (2) Diameters [basilar artery (BA) diameter and posterior cerebral artery (PCA) diameters (D1, D2)] were measured using the “Distance” tool at the midpoint of each vessel segment, perpendicular to its long axis. (3) The aneurysm neck was measured as the shortest distance across the aneurysm orifice using the “Distance” tool. (4) The maximum diameter, width, and height were measured using the “Caliber” and “Distance” tools, with height defined as the maximal perpendicular distance from the neck plane to the dome. (5) Volume was calculated automatically using the “Volume” tool after isolating the aneurysm sac from the parent vessel. (6) The lateral angle ratio (LSR) and diameter ratio (DR) were calculated as a3/a2 and D2/D1, respectively.

### Detection of morphological parameters

**Figure 1:**
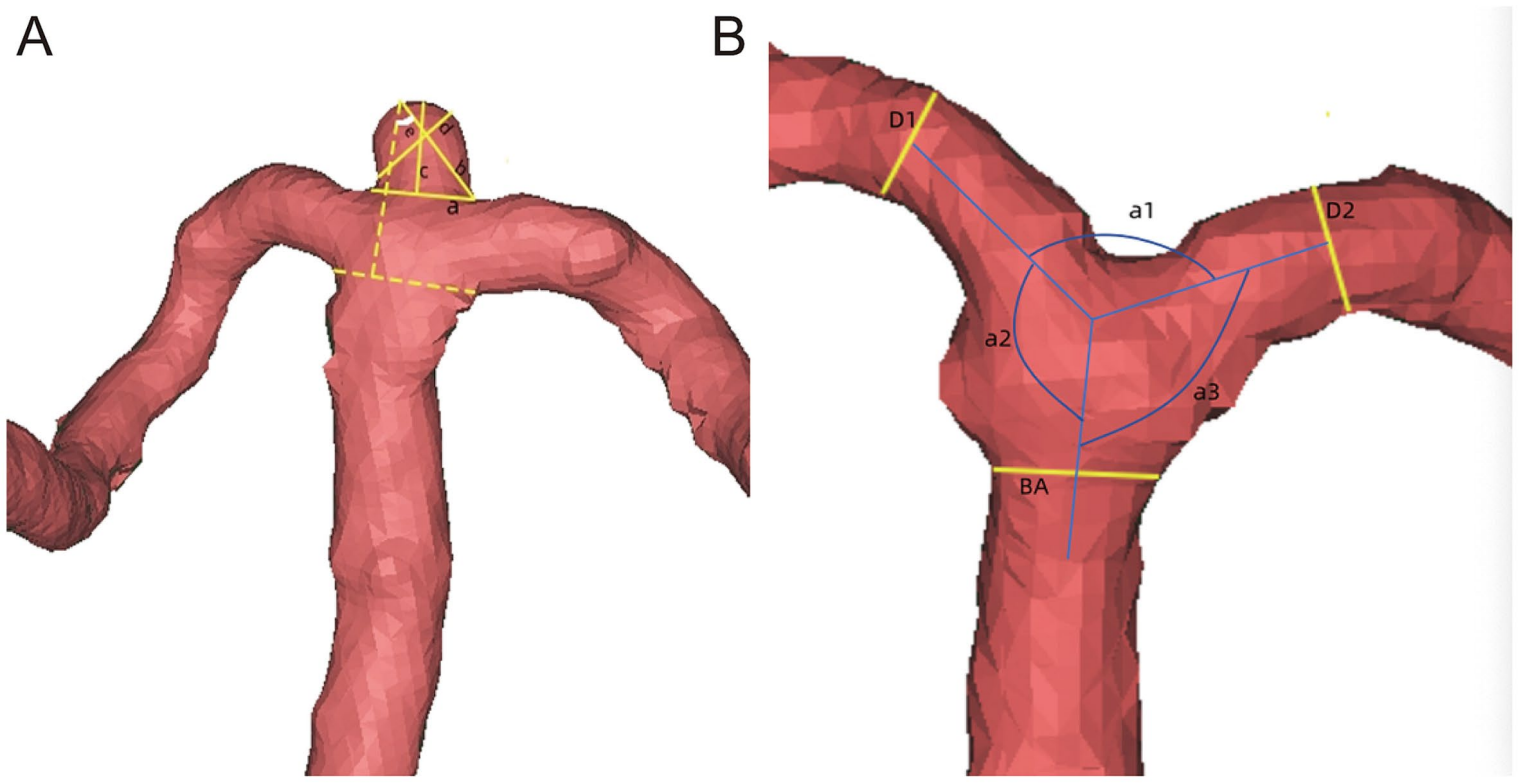
Measurements of basilar artery aneurysm morphology. **(A)** Presents various measurements related to basilar artery aneurysms, including the aneurysm neck (a), the maximum diameter of the aneurysm (b), the height of the aneurysm (c), the width of the aneurysm (d), and the angle of incidence of the aneurysm (e). **(B)** Displays additional measurements, including the diameter of the basilar artery (BA), the smaller diameter of the posterior cerebral artery (PCA) (D1), the larger diameter of the PCA (D2), the bifurcation apex angle (a1), the small lateral angle (a2), and the large lateral angle (a3) formed between the PCA and the BA.

Arterial bifurcation angles were measured using Mimics software according to the method reported by Zhang et al. (9). The bifurcation apex angle was defined as the angle formed by the posterior cerebral arteries (PCAs) on both sides (a1). The small lateral angle (LA) was defined as the smaller angle formed between the PCA and the BA (a2), and the large LA was defined as the larger angle between the PCA and the BA (a3). The LA ratio (LSR) was calculated as a3/a2. Additional measurements included aneurysm neck, maximum diameter, width, height, angle of incidence, and volume. Arterial diameters were measured according to the method reported by Ingebrigtsen et al. (17). Measurements included the BA diameter, the PCA with the smaller diameter (D1), the PCA with the larger diameter (D2), and the arterial diameter ratio (DR), calculated as D2/D1. In total, two experienced neurosurgeons, blinded to each other’s measurements and patient outcomes, independently performed all reconstructions and measurements. For each parameter, the mean value of the two measurements was used for analysis. Interobserver reliability was assessed using the intraclass correlation coefficient (ICC) with a two-way random-effects model, and all parameters showed excellent agreement (ICC > 0.90). Definitions of the measured parameters are illustrated in Figure 1.

### Variable selection method

To ensure the robustness of the prognostic model and to avoid overfitting, the number of candidate predictor parameters was determined based on the number of outcome events. A total of nine deaths were reported during the follow-up period. According to the widely recognized events per variable (EPV) criterion, a minimum of 10 events per predictor parameter is recommended to maintain model stability and reduce overfitting (18). Based on this criterion, the maximum number of predictor parameters that could be reliably evaluated was less than 1. Therefore, a conservative modeling strategy was adopted. Variables were first screened using the least absolute shrinkage and selection operator (LASSO)-penalized Cox regression to reduce dimensionality, and only predictors with non-zero coefficients were considered for the final multivariable Cox regression model. This approach aligns with recent methodological recommendations for developing prediction models in studies with limited sample sizes (18, 19).

### Statistical analysis

The least absolute shrinkage and selection operator (LASSO)-penalized Cox regression analysis was performed for variable selection and prognostic model development using the R package “glmnet” v4.1–8. The optimal penalty parameter (*λ*) was determined via tenfold cross-validation, with lambda.min selected as the tuning parameter. Variables with non-zero coefficients at this λ value were retained, yielding two key prognostic variables. A multivariable Cox proportional hazards regression model was subsequently constructed using the R package “survival” version 3.7.0. A nomogram was generated using the “rms” package (v6.8.1) for visualization, and model performance was assessed using time-dependent calibration curves (1-, 2-, and 3-year) based on the Kaplan–Meier method with bootstrap resampling (B = 200). The nomogram was deployed as a web-based tool to enhance usability. Model performance was evaluated in terms of discrimination and calibration. The concordance index (C-index) and the receiver operating characteristic (ROC) curves were used to assess the model’s discriminatory power. Bootstrap resampling was applied for internal validation, and calibration curves were generated to illustrate the agreement between the observed and predicted outcomes. Decision curve analysis (DCA) was conducted to evaluate the model’s clinical utility. A *p*-value of < 0.05 was considered statistically significant.

The dynamic nomogram was deployed as an interactive web application using the shiny package (version 1.7.4) in R (version 4.2.0). The application is hosted on the shinyapps.io cloud platform (RStudio, Boston, MA, United States), which provides automated load balancing and HTTPS encryption. The user interface allows manual input of two continuous variables: Basilar artery diameter and aneurysm neck. Predicted survival probabilities are calculated in real time based on the fitted Cox proportional hazards model, using the baseline cumulative hazard function estimated from the study cohort.

Importantly, the web application is designed with a no-data-storage policy: No user-input data are stored on the server, and all entered values are discarded immediately after the prediction is generated. The application does not collect any personally identifiable information (e.g., name, medical record number, or date of birth). Communication between the user’s browser and the server is secured via HTTPS. A disclaimer is displayed on the application interface stating that the tool is intended for research and educational purposes only and does not replace clinical judgment. The application is freely accessible at https://wang97.shinyapps.io/dynnomapp/, and the underlying R code is available from the corresponding author upon reasonable request.

## Results

### Demographics

A total of 58 patients were included and followed up for this study. The mean age was 63.66 years, with a higher prevalence among female individuals. A total of 14 patients (24.14%) had a history of hypertension, 3 (5.17%) had diabetes, 15 (25.86%) were smokers, and 5 (8.62%) reported alcohol consumption. Regarding arterial bifurcation angles, 43.10% of patients had an angle a1 greater than the mean value of 149.75°, 56.90% had an angle a2 greater than 88.63°, and 58.62% had an angle a3 greater than 121.63°. For other vascular parameters, 20.69% of patients had an LSR above the mean value of 1.55, 41.38% had D1 greater than 1.95, 43.10% had D2 greater than 2.56, and 34.48% had a BA diameter greater than 3.56. The DR exceeded the mean value of 0.77 in 51.72% of cases. In terms of aneurysm characteristics, 27.59% of patients had a neck width greater than the mean value of 6.07 mm, 37.93% had a maximum diameter greater than 5.11 mm, 39.65% had an aneurysm width greater than 4.93 mm, 39.66% had a height greater than 4.63 mm, 43.10% had incidence angularity greater than 63.52°, and 20.69% had a volume greater than 28.84 (Table 1). The median follow-up duration was 17.5 months (range, 1–58 months).

### Model development and variable selection

Among the 58 included patients, 9 (15.5%) died during the overall follow-up period. Given the limited number of outcome events, a conservative modeling strategy was adopted to avoid overfitting. According to methodological recommendations, an EPV ratio of at least 10 is generally required to ensure model stability. With only nine events, the maximum number of predictor parameters that could be reliably evaluated was less than 1. Therefore, the variables were first screened using LASSO-penalized Cox regression to reduce dimensionality and select the most relevant predictors.

### Development of the nomogram

**Figure 2:**
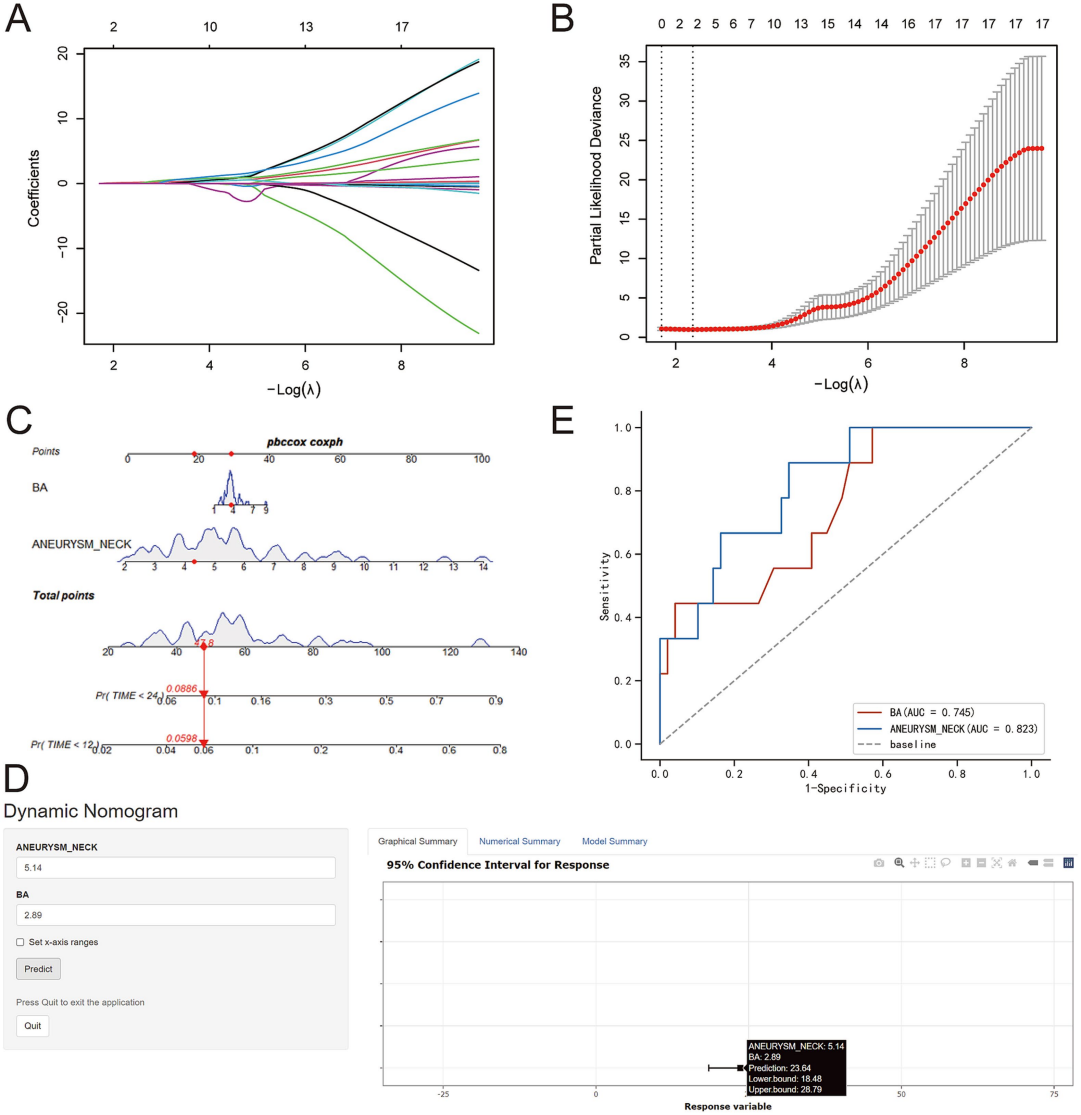
Integrated Prognostic Evaluation for Basilar Artery Aneurysm Embolization: Nomogram, Dynamic Survival Curves, and ROC Analysis **(A)**. LASSO-penalized Cox regression coefficient profiles. The vertical axis shows the regression coefficient values for each candidate predictor, and the horizontal axis represents –Log (*λ*). The optimal penalty parameter λ was selected via ten-fold cross-validation (lambda.min), with the vertical dashed line indicating the selected λ value. **(B)** Cross-validation curve for LASSO-penalized Cox regression. The partial likelihood deviance is plotted against –Log (λ). The vertical dashed line indicates the optimal λ (lambda.min) that minimizes the cross-validated deviance, resulting in the retention of two predictors with non-zero coefficients. **(C)** A nomogram was developed by combining two parameters: Basilar artery (BA) diameter and aneurysm neck. Points were assigned according to each parameter value. The total points represent the sum of the points assigned after considering all parameters. Pr(TIME<12), one-year survival; Pr(TIME<24), two-year survival. **(D)** Both the BA diameter and aneurysm neck were identified as risk factors predicting survival. **(E)** ROC curve used to evaluate the model’s discriminative ability.

Based on the LASSO-penalized Cox regression, two variables—BA diameter and aneurysm neck—were identified as independent risk factors associated with survival (Figures 2A,B). The Cox proportional hazards regression analysis further confirmed that BA diameter (HR = 2.12, 95% CI: 1.01–4.45, *p* = 0.047) and aneurysm neck (HR = 2.29, 95% CI: 1.04–6.23, *p* = 0.003) were significant predictors of survival after endovascular embolization of basilar artery aneurysms (Table 2). To visualize the predictive results and facilitate clinical application, a nomogram was developed using R software (version 4.2.0) (Figures 2C,D). In addition, the model demonstrated good discriminatory performance, with an area under the ROC curve of 0.745 for BA diameter and 0.823 for aneurysm neck (Figure 2E).

**Table 2:** Univariate and multivariate Cox risk proportional regression analyses in patients undergoing interventional embolization of basilar aneurysms.

Variables	Univariate analysis	Multivariate analysis
	HR (95% CI) *p*-value	HR (95% CI) *p*-value
Age	1.02 (0.96–1.08) 0.543	
Sex	0.89 (0.28–4.52) 0.863	
Hypertension	0.43(0.63–8.72) 0.203	
Diabetes	2.98(0.37–23.98) 0.304	
Smoking	0.82(0.17–3.96) 0.807	
Alcohol abuse	1.63(0.20–13.13) 0.648	
Angle of arterial bifurcation		
a1	1.00 (0.99–1.01) 0.732	
a2	1.00 (0.97–1.02) 0.698	
a3	0.99 (0.98–1.02) 0.889	
LSR	0.87 (0.39–1.93) 0.736	
Vessel diameter		
D1	1.16 (0.65–2.06) 0.613	
D2	1.18 (0.69–2.02) 0.539	
BA	1.55 (1.16–2.09) 0.003	2.12 (1.01–4.45) 0.047
DR	0.48 (0.01–18.22) 0.695	
Arterial aneurysm parameters		
Aneurysm neck	1.42 (1.16–1.72) < 0.001	2.99 (1.44–6.23) 0.003
Maximum diameter	1.14 (0.96–1.35) 0.134	0.37 (0.15–0.90) 0.028
Width	1.04 (0.90–1.21) 0.598	
Height	1.17 (0.93–1.48) 0.181	1.14 (0.59–2.10) 0.697
Incidence angle	1.00 (0.99–1.02) 0.374	
Volume	0.99 (0.99–1.02) 0.466	

### Validation of the nomogram

**Figure 3:**
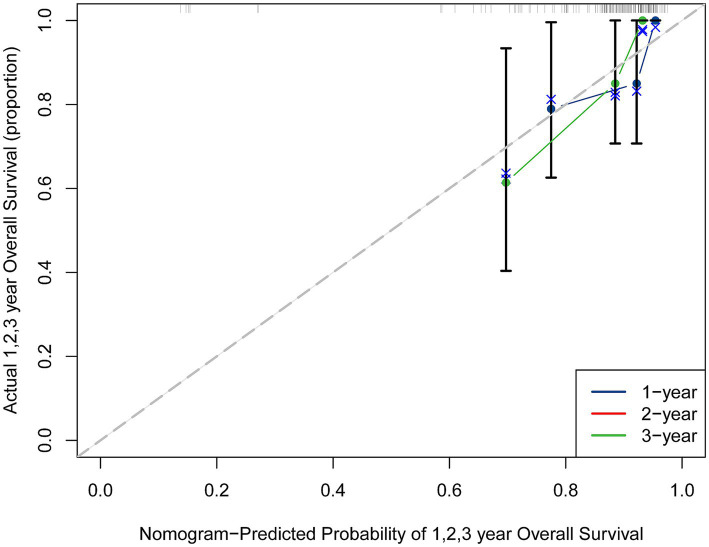
Time-dependent calibration curves for the prognostic nomogram. Calibration curves for predicting survival at 1 year (left panel), 2 years (middle panel), and 3 years (right panel) after endovascular embolization.

Internal validation of the developed model was performed using bootstrap resampling. Time-dependent calibration curves (Figure 3) demonstrated close agreement between the predicted and observed survival rates in patients undergoing endovascular embolization for basilar artery aneurysms, indicating the model’s satisfactory predictive accuracy.

### Clinical utility assessment using decision curve analysis (DCA)

**Figure 4:**
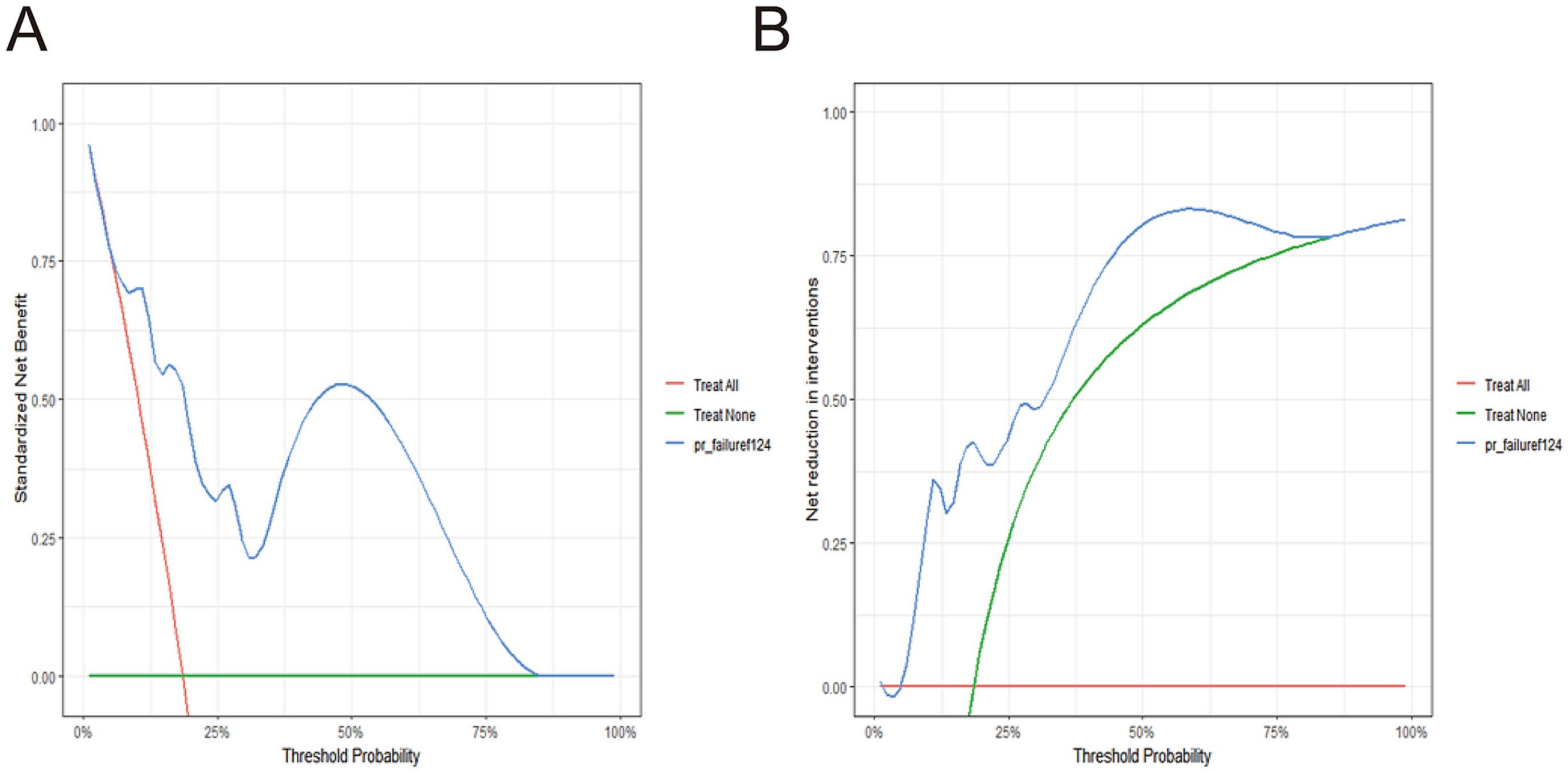
Decision curve analysis of the nomogram for predicting survival after basilar artery aneurysm endovascular embolization in patients undergoing the procedure. **(A)** When the high-risk threshold probability was between 0 and 100%, the model yielded a greater net benefit compared to the “treat-all” (performing embolization for all patients) and “treat-none” (performing embolization for no patients) strategies. **(B)** The x-axis represents the net reduction in unsuccessful basilar artery aneurysm endovascular embolizations per 100 patients. DCA showed that the model achieved a net reduction of 80 unsuccessful cases at a decision threshold above 85%.

The DCA was conducted to evaluate the clinical utility of the developed prognostic model. The threshold probability represents the minimum probability of an adverse outcome (e.g., death during the follow-up period) at which a clinician would consider performing endovascular embolization, balancing the potential benefits against the risks and harms of the procedure. As shown in Figure 4A, across threshold probabilities ranging from 0 to 100%, the model consistently demonstrated a higher net benefit compared to the “treat-all” (performing embolization for all patients) and “treat-none” (performing embolization for no patients) strategies, indicating that the model provides clinical value across a wide range of risk tolerance levels.

The net benefit was further quantified as the reduction in net harm relative to a reference strategy. Specifically, at a threshold probability of 85%, the model achieved a net reduction of more than 80 unsuccessful cases per 100 patients compared to the “treat-all” strategy (Figure 4B). This indicates that, by applying the model to guide treatment decisions, clinicians can avoid approximately 80 unnecessary or potentially unsuccessful embolization procedures per 100 patients. Even at a high decision threshold of >90%, the model continued to demonstrate a positive net benefit, supporting its reliability even when clinicians require a very high level of certainty before proceeding with intervention.

## Discussion

Vascular morphology, hemodynamics, hypertension, sex, age, smoking, and alcohol use are all risk factors that contribute to the development and progression of aneurysms. Among these, vascular morphologic factors play an important role in the initiation, formation, and rupture of aneurysms (20–22). There is an interplay between aneurysm geometry, hemodynamics, and pathobiology, with geometry determining the status of blood flow, which in turn influences the remodeling and growth of the aneurysm through pathobiology, thereby altering its geometry (23–26). Previous studies have shown significant differences in the middle cerebral artery (MCA), internal carotid artery (ICA), and basilar artery (BA) between patients with and without aneurysms (27, 28). Previous findings have noted that patients with basilar artery aneurysms have significantly (*p* < 0.05) reduced bifurcation and branch vessel angles compared to controls (28). Previous studies have also demonstrated that, according to Laplace’s law, arteries with thinner walls experience greater wall tension under the same blood pressure (29). Consequently, for two aneurysms of equal size, the smaller the diameter of the artery carrying the aneurysm, the greater the tension in the aneurysm wall. In addition, variability in aneurysm morphology, size, and location contributes to different rupture risks (30). Furthermore, the enlargement of the aneurysm neck is a significant risk factor for rupture and is strongly associated with patient survival time. A correlation between basilar artery (BA) diameter, aneurysm neck, and patient prognosis has been established in previous studies, which aligns with the findings of this study’s Cox analysis. Therefore, the present study selected morphological parameters related to aneurysms based on previous literature to develop a novel and practical system for predicting survival after endovascular embolization in patients with BA aneurysms.

We constructed a prediction model using the R language and compared it to traditional statistical software (SPSS and Statistica). The experimental design employed is more innovative, the techniques used are more comprehensive, and the results are more accurate. According to previous studies, the effectiveness of endovascular embolization as a treatment for basilar artery aneurysms has been well-established (31). At the same time, previous studies have shown correlations between BA aneurysm formation and older age, female sex, a wider apical angle of arterial bifurcation, parent vessel diameter, and aneurysm morphological characteristics (9). In the present study, Cox survival analysis also showed that BA diameter and aneurysm neck were associated with prognosis after endovascular embolization in patients with basilar artery aneurysms. Other factors, including sex, age, a1, a2, a3, D1, D2, DR, LSR, maximum aneurysm diameter, width, height, incidence angle, and volume, were not significantly associated with prognosis after endovascular embolization of basilar artery aneurysms. Our findings align with recent advances in neurovascular predictive modeling. Several studies published between 2023 and 2025 have employed radiomics, machine learning, and deep learning approaches to predict intracranial aneurysm rupture risk and treatment outcomes (10–12). However, these models primarily focus on rupture risk prediction rather than post-interventional survival, and few of these models have been deployed as user-friendly web-based tools for real-time clinical application. Our study addresses this gap by providing an easily accessible online nomogram specifically designed for survival prediction after endovascular embolization. Therefore, we developed an online interactive nomogram based on the relevant risk factors identified above. The C-index and ROC analyses were mainly used to assess the discriminative ability of the model. Bootstrap resampling was used for internal validation, and we constructed calibration curves to evaluate the model and assessed the clinical applicability of the model using the DCA. All of the above metrics indicated that the constructed model demonstrated good discrimination, calibration, and clinical applicability. In practical clinical application, individual postoperative survival predictions can be rapidly obtained by simply inputting patient data into the online version of the model. When the high-risk threshold probability was between 0 and 100%, the current prediction model showed a higher net benefit compared to both the “treat-all” strategy (treating all patients) and the “treat-none” strategy (treating no patients). In addition, the current model reduces the risk of unsuccessful outcomes by more than 85% and may still provide clinical value even when clinicians estimate the probability of success to exceed 90%.

Nomograms are currently used to predict prognosis in patients with brain injury and cancer and to replace traditional prediction models (22, 32–34). In a study by Zhang et al. (7), prognosis was mainly analyzed by combining preoperative imaging and vascular morphology or hemodynamic parameters or by developing a static model to predict patient outcomes. In this study, we constructed a three-dimensional model based on preoperative imaging to quantify morphological features related to basilar artery aneurysms and developed a novel predictive model using R. The aim was to assist clinicians in selecting the optimal surgical approach preoperatively, provide more valuable guidance, and reduce the incidence of unnecessary procedures (6, 8, 35). We developed a web-based version of the prediction model to facilitate clinical use and enable more timely and accurate risk estimation. As shown in Figure 3,^1^ predictions can be made by entering individualized patient information. For example, in a patient with a basilar artery aneurysm measuring 2.89 in diameter and an aneurysm neck of 5.14, the model generates a predicted value of 23.64, indicating a relatively long post-treatment survival and supporting a favorable prognosis. The system is simple to use, provides timely and accurate predictions, and is easy to understand.

The current study has several limitations. First, it was conducted at a single center with a relatively small sample size, which may introduce selection bias. Second, the study did not elucidate whether geographic, hemodynamic, or other individualized factors influence patients’ postoperative survival (36, 37). Future studies should expand the sample size and incorporate other possible relevant risk factors to improve model performance, and multicenter studies should be conducted to enable external validation using data from other centers, with randomly selected patients with BA aneurysms. Previous studies have shown that factors such as hypertension, diabetes mellitus, smoking history, and alcohol consumption are involved in the development of aneurysms (20–22). In addition, only nine outcome events occurred during the overall follow-up period. According to methodological recommendations for clinical prediction models, an EPV ratio of at least 10 is generally required to ensure model stability and avoid overfitting (18). The limited number of outcome events restricted the number of predictor parameters that could be reliably included in the multivariable Cox model. To mitigate the risk of overfitting, we adopted a conservative variable selection strategy using LASSO-penalized Cox regression, a method that has also been applied in prognostic studies with limited sample sizes (19). Nevertheless, the results should be interpreted as exploratory, and external validation in larger, independent cohorts is warranted. Third, although previous studies have suggested that factors such as hypertension, diabetes mellitus, smoking history, and alcohol use may contribute to aneurysm development, these variables were not identified as significant predictors of survival in our analysis. This may be attributable to the limited sample size and number of outcome events. In addition, this was a retrospective cohort study, and follow-up bias is therefore unavoidable. Physicians should also consider other risk factors related to patients’ overall health status, comorbidities, and preferences when determining the appropriate treatment plan. Finally, the follow-up period in this study was limited to a median of 17.5 months; longer follow-up is needed to validate the accuracy of the prognostic model in future studies.

## Conclusion

We developed an online dynamic nomogram incorporating basilar artery diameter and aneurysm neck morphology to predict survival after endovascular embolization of unruptured basilar artery aneurysms. The model demonstrated acceptable discrimination (C-index = 0.805), good calibration, and favorable clinical utility across a range of threshold probabilities, as assessed by decision curve analysis.

Despite the limitations of a small, single-center retrospective study, this work provides a novel web-based tool that integrates easily measurable morphological parameters to support individualized survival prediction. The nomogram offers clinicians a practical and accessible aid for risk assessment and shared decision-making in patients undergoing endovascular embolization. External validation in larger, multicenter cohorts is warranted to further confirm its generalizability.

## Data Availability

The original contributions presented in the study are included in the article/supplementary material, further inquiries can be directed to the corresponding author/s.

## Footnotes

1 Available at: https://wang97.shinyapps.io/dynnomapp/
